# Comparison of computed tomographic ocular biometry in brachycephalic and non-brachycephalic cats

**DOI:** 10.14202/vetworld.2021.727-733

**Published:** 2021-03-23

**Authors:** Kittiporn Yuwatanakorn, Chutimon Thanaboonnipat, Nalinee Tuntivanich, Damri Darawiroj, Nan Choisunirachon

**Affiliations:** 1 Department of Veterinary Surgery, Faculty of Veterinary Science, Chulalongkorn University, Bangkok, Thailand; 2 Department of Veterinary Anatomy, Faculty of Veterinary Science, Chulalongkorn University, Bangkok, Thailand

**Keywords:** age, biometry, cat, computed tomography, eye, skull

## Abstract

**Background and Aim:**

Ocular biometry has been used to evaluate ocular parameters; however, several factors need to be considered. In humans, age and sex have been shown to affect ocular biometry. The main factor that affects feline ocular biometry is the head circumference. At present, several reports have revealed that canine ocular biometry differs among dog breeds. However, there are no reports on normal ocular biometry in cats using computed tomography (CT). Therefore, this study aimed to explore feline ocular parameters between brachycephalic (B) and non-brachycephalic (NB) cats using CT and to evaluate the influence of age or sex of cats on ocular biometry.

**Materials and Methods:**

Twenty-four normal cats were divided into two groups: B (n=12) and NB (n=12). Each group had an equal number of designated males and females. CT was performed under mechanical restraint without general anesthesia and intravenous contrast enhancement. Ocular biometry, dimensions of the internal structure, including attenuation numbers and extra-ocular structures, were evaluated and compared.

**Results:**

B-cats had a significantly wider globe width (GW) than NB-cats (p<0.05). In addition, globe length (GL) and GW were significantly correlated with the age of the cats. Significant correlation between GL and age was observed in all cats (r=0.4867; p<0.05), NB-cats (r=0.8692; p<0.05), and B-cats (r=0.4367; p<0.05), whereas the correlation between GW and age was observed in B-cats only (r=0.7251; p<0.05). For extra-ocular structures, NB-cats had significantly greater orbital depth than B-cats (p<0.05), and orbital diameter was significantly correlated with age in all cats and B-cats (p<0.05).

**Conclusion:**

CT can be used for ocular biometric evaluation in cats with different skull types. GW was wider in B-cats, whereas the orbital depth was greater in NB-cats. Moreover, GW, GL, and orbital diameter were affected by the age of the cats. This information will be useful for further ocular diagnosis and treatment, especially in prosthetic surgical procedures.

## Introduction

The eye is a vital sensory organ necessary for animals to have a good quality of life because it enables vision. The eye includes a spherical soft tissue structure, called the globe, and adjacent soft-tissue structures. In addition, the orbit, an incomplete bony fossa in carnivores, is an area embedded with ocular anatomical structures. Ocular diseases in dogs and cats can be divided into several types according to the affected anatomical areas. Most dogs and cats are affected by corneal disease [1,2]. However, ocular and periocular abnormalities that cause structural changes have been frequently reported. In cats, several diseases cause blindness, one of which is retrobulbar neoplasia, which was reported to account for 4% of neoplasm in dogs and cats [3]. According to the location, the detection of retrobulbar abnormalities might be difficult. Most owners and veterinarians frequently neglect or misdiagnose them. To evaluate the eye objectively, several imaging modalities have been applied: Ultrasonography (US) [4], computed tomography (CT) [5], and magnetic resonance imaging (MRI) [6]. The US is a radiation-free imaging modality that entails a lower cost than CT and MRI. However, assessment of an adjacent structural alteration induced ocular displacement on US is difficult, as observed for diseases of the orbit or retrobulbar masses. Recently, CT has become a novel diagnostic method in veterinary medicine, increasingly being applied worldwide, especially in Thailand. CT can reveal multidirectional images of anatomical structures for ocular biometry. Moreover, CT is superior to US and MRI, as it reveals bony changes around the ocular area as well.

Although there is abundant information on mammalian eyes, various factors need to be considered before diagnosis. Age and sex can affect the human ocular dimensions [7]. In addition, in dogs, body weight should be considered as a factor for determining ocular dimensions [8]. In contrast to dogs, the main factor that affects feline ocular biometry ultrasonographically is head circumference [9]. At present, CT is one of the most sensitive diagnostic imaging modalities. CT provides useful morphological and topographic data and is presently pronounced as a diagnostic imaging modality of choice for the head and neck areas. CT provides volumetric data of the eye, including the periocular and retrobulbar areas of the patient. However, several anatomical variations must be considered before the final diagnosis.

Therefore, this study was aimed to preliminarily investigate the normal variation in ocular biometry on CT between non-brachycephalic (NB) and brachycephalic (B) cats.

## Materials and Methods

### Ethical approval and informed consent

This cross-sectional study was approved by the Institutional Animal Care and Use Committee of Chulalongkorn University (CU-IACUC; 1831094).Written consents were obtained from all cat owners. All cats in the current study were attended within the same day and were allowed to return home after the study.

### Study location and period

This study was designed as the prospective study performed on cats presenting to the Small Animal Hospital, Faculty of Veterinary Science, Chulalongkorn University from January to June 2019.

### Animals and experimental design

#### Animals

Cats were divided into two groups, B cats and NB, comprising 12 in each group according to skull type, categorized by the criteria of the Cat Fanciers’ Association. An equal number of male (n=6) and female (n=6) cats were assigned to each group. Information such as the skull type, age, and sex of each cat was obtained and recorded. Cats that showed any signs of ophthalmic or orbital diseases, including abnormalities related to the adjacent orbital area, were excluded from the study. Before the CT scan, all selected cats were ensured to be healthy through physical examination, neurological examination, and basic ophthalmic examinations, including intraocular pressure (IOP) and fluorescein staining test.

#### CT procedure

In this study, for the CT procedure, cats were non-sedated and mechanically restrained using a caring box supported by rolled towels. The rolled towels helped minimize movement and provided a suitable and comfortable position for all cats. The number of rolled towels inserted into the box varied depending on the size of the individual cat.

Each cat was positioned in sternal recumbency with the direction of the head pointing to the CT gantry, while the skull was perpendicular to the isocenter of the CT scan planes. Non-contrast-enhanced CT images were acquired using a 64-slice helical CT unit (Optima CT660^®^, GE, Japan). The technical settings were 120 kVp and the automated mA. The effective slice thickness was 0.625 mm, the collimator pitch 0.935 mm, and the matrix size 512×512 (isotropic voxels). CT images served as Imaging and Communications in Medicine (DICOM) files and were processed by the image viewer using Osirix^®^ software (Osirix^®^, Geneva, Switzerland) on a non-CT unit work station with a monitor matrix size of 2560×1440. Images were analyzed by soft-tissue window using 350 Hounsfield units (HU) of the window width and 40 HU of the window level.

### Analytical procedure

#### Ocular biometric values

**Figure-1:**
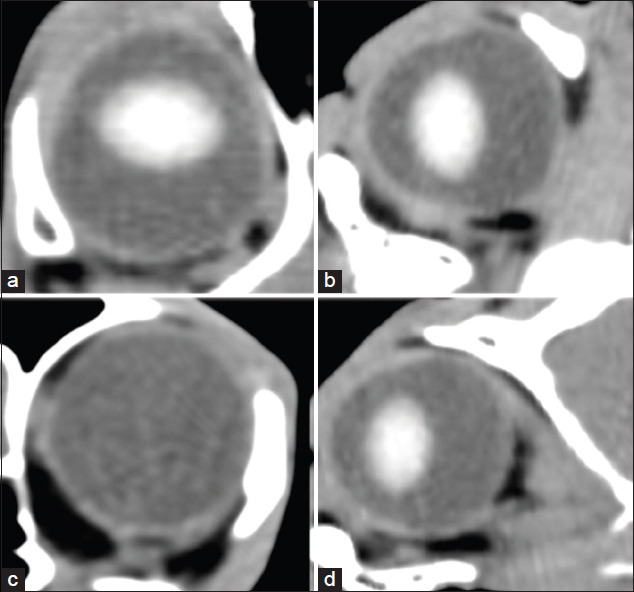
Multiplanar reconstructed-computed tomographic images on each plane; dorsal plane (a), sagittal plane (b), axial plane (c), sagittal oblique to reveal the most depth of retrobulbar region (d) for measurement globe length (a and b), globe width (a and c), globe height (b and c), anterior chamber depth (a and b), lens thickness (a and b), vitreous chamber depth (a and b), orbital depth (d), orbital diameter (d), and retrobulbar depth (d).

To measure the actual dimensions of the eye, multiplanar reconstruction was applied to obtain the proper axes of the globe in each direction. All parameters were then measured using an in-built digital caliper. All ocular dimensions were recorded as follows: (1) Globe length (GL) measured on horizontal and sagittal planes from the maximal distance of the corneal epithelium to the fundus, (2) globe width (GW) measured on horizontal and equatorial planes from the maximal distance of the medial to the lateral aspect of the globe fundus, (3) globe height (GH) measured on sagittal and equatorial planes from the maximal distance of the retina-choroid-sclera complex to the retina-choroid-sclera complex, (4) anterior chamber depth (ACD) measured on the sagittal and horizontal planes from the maximal distance of the corneal endothelium to the anterior lens capsule, (5) lens thickness (LT) measured on the sagittal and horizontal planes from the maximal distance of the axial point of the anterior to the posterior lens capsule, (6) vitreous chamber depth (VCD) measured on the sagittal and horizontal planes from the maximal distance of the axial point of the posterior lens capsule to the fundus and on the sagittal oblique plane that showed the deepest retrobulbar region, (7) orbital depth measured from the maximal distance of the most anterior of the orbit and the posterior of orbit, (8) orbital diameter measured from the maximal distance of the medial orbital rim to the lateral orbital rim, and (9) retrobulbar depth (RD) measured from the maximal distance of the most posterior of the eye ball and the deepest of the retrobulbar region (Figure-1).

#### Intraocular CT attenuation numbers

The CT attenuation numbers were measured by drawing a region of interest (ROI) over the center of the anterior chamber, lens, and vitreous chamber with an area of 0.02 cm^2^ and reported as attenuation numbers (HU).

### Statistical analysis

All data in this study were expressed as descriptive data. The GL, GW, GH, ACD, LT, and VCD measured from two planes in each animal were averaged. Each ocular biometric value, such as GL, GW, GH, ACD, LT, and VCD, in all and each group of healthy cats were presented as means with standard deviation (including the minimal and maximal values). The differences in parameters between the groups were tested using an unpaired t-test. Relationships and associations between age and sex were assessed using linear regression analysis and Pearson’s coefficient test, respectively. All statistical analyses were considered significant if p<0.05.

## Results

### Clinical demographic data

The NB group included 12 domestic shorthair breed cats, whereas the B-cats included American shorthair (n=4), Persian (n=3), Scottish Fold and mix breed (n=2 each breed), and British shorthair (n=1) cats. All the female cats were neutered. Among the male cats, there were two intact cats and ten castrated cats. The gonadal status of the cats in each group, including age and body weight, is shown in Table-1.

**Table-1:** Clinical demographic information of non-brachycephalic and brachycephalic cats.

Clinical features	Value
No. of patients	24
Brachycephalic	12
Non-brachycephalic	12
Age (months)	
All cats	
Median	36.00
Mean±SD	39.54±12.44
Range	(20.00-60.00)
Non-brachycephalic	
Median	42.00
Mean±SD	40.00±14.00
Range	(20.00-60.00)
Brachycephalic	
Median	36.00
Mean±SD	35.00±9.11
Range	(24.00-48.00)
Gender	
All cats	
Female	
Intact	2
Spayed	10
Male	
Intact	0
Castrated	12
Non-brachycephalic	
Female	
Intact	1
Spayed	5
Male	
Intact	0
Castrated	6
Brachycephalic	
Female	
Intact	1
Spayed	5
Male	
Intact	0
Castrated	6

#### Computed tomographic information of the eye

Globe size

**Figure-2:**
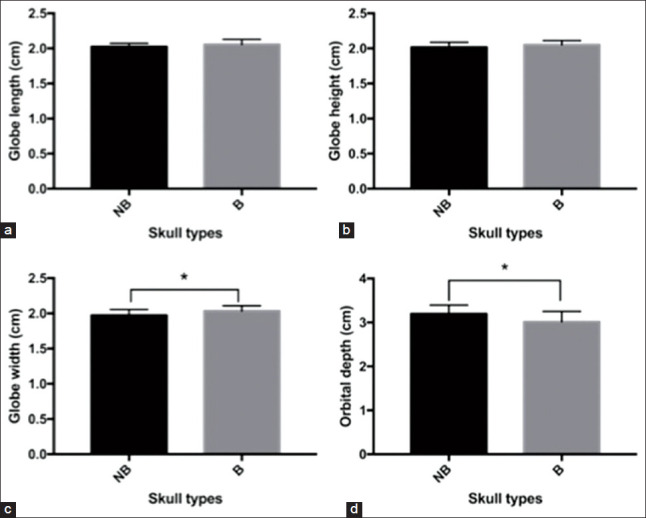
Globe size in each dimension; globe length (a), globe height (b), globe width (c) and orbital depth (d) between non-brachycephalic (NB) and brachycephalic (B) cats. Globe width of the B cats was significantly wider than that of the NB cats and orbital depth of the NB cats was significantly deeper than that of the B cats.

All measurements of globe size are presented in Table-2. No statistically significant difference was found for GL and GH between the left and right eyes, plane orientations, and skull types (Figure-2a and b). Although a significant difference was not found for GW between the left and right eyes and plane orientations, the skull type had an effect on GW. The GW of B-cats was significantly wider than that of NB-cats (p<0.05; Figure-2c).

**Table-2:** Ocular biometric data and Hounsfield unit (mean±SD; cm and HU) of globe size, internal structures, and external structures between right (ocular dextrus: OD) and left (ocular sinister: OS) eyes in non-brachycephalic and brachycephalic cats.

	All cats		Non-brachycephalic		Brachycephalic	
		
	OD	OS	OD	OS	OD	OS
Globe size						
GL	2.03±0.04	2.03±0.05	2.02±0.03	2.02±0.04	2.04±0.05	2.02±0.04
GW	2.00±0.08	1.98±0.07	1.97±0.07	1.98±0.06	2.02±0.04*	1.99±0.08*
GH	2.02±0.05	2.02±0.05	2.04±0.08	2.01±0.06	2.02±0.05	2.02±0.05
Internal structures						
ACD	0.37±0.03	0.37±0.04	0.36±0.03	0.36±0.04	0.37±0.04	0.37±0.04
ACD (HU)	12.48±3.72	11.87±3.23	12.55±4.24	12.19±3.37	12.40±3.30	12.40±3.30
LT	0.85±0.03	0.85±0.03	0.85±0.03	0.85±0.03	0.87±0.02	0.87±0.04
LT (HU)	154.9±7.54	152.4±11.31	154.7±7.85	152.2±12.67	155.01±7.56	152.5±10.48
VCD	0.76±0.04	0.74±0.04	0.76±0.04	0.75±0.03	0.76±0.04	0.74±0.04
VCD (HU)	15.40±4.11	14.92±3.52	15.44±4.01	15.04±3.41	15.36±4.93	14.51±3.76
External structures						
Orbital depth	3.09±0.23	3.12±0.25	3.18±0.20	3.21±0.19	3.01±0.24*	3.03±0.26*
Orbital diameter	2.68±0.16	2.65±0.17	2.65±0.17	2.63±0.17	2.70±0.15	2.68±0.18
RD	1.77±0.19	1.78±0.19	1.78±0.15	1.84±0.22	1.63±0.10	1.58±0.12

Statistically difference of averaged eye parameters between non-brachycephalic and brachycephalic cat groups was made using Unpaired t-test,

* p<0.05. GL=Globe length, GW=Globe width, GH=Globe height, ACD=Anterior chamber depth, LT=Lens thickness, VCD=Vitreous chamber depth, RD=Retrobulbar depth, HU=Hounsfield unit, OS=Oculus sinister, OD=Oculus dextrus

**Figure-3:**
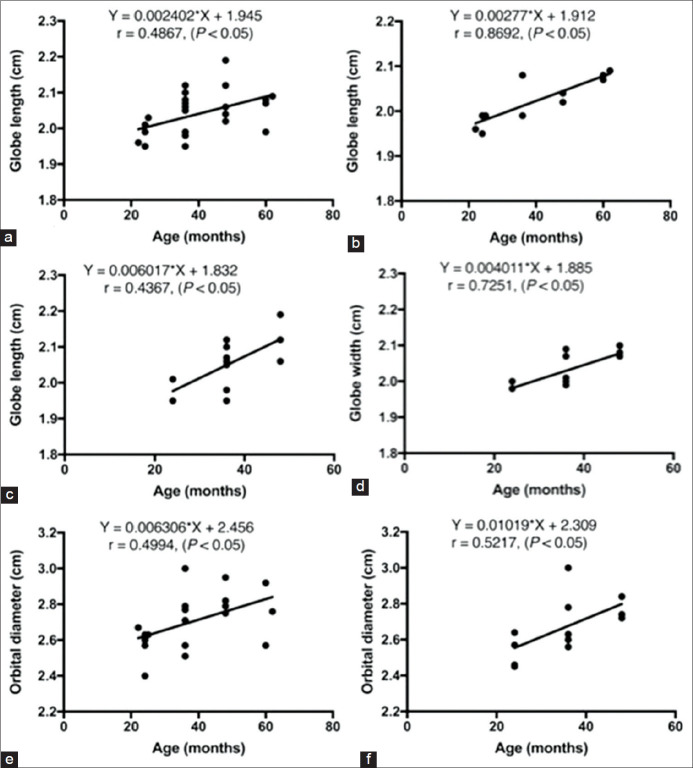
The correlations between ocular structures and age; globe length and age in all cats (a), globe length and age in non-brachycephalic cats (b), globe length and age in brachycephalic cats (c), globe width and age in brachycephalic cats (d), orbital diameter and age in all cats (e), and orbital diameter and age in brachycephalic cats (f).

A significant correlation was found between GL and age of all cats (r=0.4867), (p<0.05) (Figure-3a), NB-cats (r=0.8692), (p<0.05) (Figure-3b), and B-cats (r=0.4367) (p<0.05) (Figure-3c). In addition, a significant correlation was found between GW and age in B-cats (r=0.7251) (p<0.05) (Figure-3d), but it was not detected in other groups.

#### Internal structures of eye

All measurements of the internal structural parameters, including attenuation numbers (HU) of each structure, are reported in Table-2. The ACD, LT, and VCD did not show statistically significant difference between the left and right eyes, plane orientations, and skull types, including the attenuation number. In addition, considering the age of the cats, no statistically significant correlation was found in any group.

#### External structures of eyes

All measurements of the orbital depth, orbital diameter, and RD are reported in Table-2. No statistically significant differences were found for orbital depth, orbital diameter, and RD between the left and right eyes. However, a significant difference was found in the orbital depth between B- and NB-cats (p<0.05) (Figure-2d). NB-cats had a significantly deeper orbit than B-cats. Considering the age of the cats, a significant correlation was found for orbital diameter in all cats (r=0.4994), (p<0.05) (Figure-3e), and B-cats (r=0.5217) (p<0.05) (Figure-3f). The older cats in all cats and B-cats group had a significantly larger orbital diameter than younger cats.

## Discussion

Ocular biometry is useful for ocular diagnosis, especially for congenital anomalies such as macrophthalmia, microphthalmia, and anophthalmia. To perform surgical correction in these cases or other acquired abnormalities, cosmetic prosthetic eyes are sometimes necessary. In addition, cosmetic prosthesis replacement is important to prevent the collapse of orbital bony structures, which leads to facial deformity [10]. In humans, complaints due to discomfort concurrent with poor fit-prosthetic eyes have been reported [11]. Other clinical signs that could be found concurrently with oversized ocular prosthesis are mucoid discharge, swollen lids, hyperemic palpebral conjunctiva, entropion on the upper and lower lid with madarosis at the nasal portion of the lower eyelid [11], and rare conjunctival squamous cell carcinoma [12]. In addition, further complications such as loose prosthesis could be the cause of giant papillary conjunctivitis, blepharoconjunctivitis sicca [13], and granuloma formation in the eye [14]. To achieve an accurate prosthesis size for each individual patient, proper diagnostic images to elucidate the precise globe dimension are important.

US is a non-invasive technique. It is a radiation-free, safe, and quick technique that normally does not require sedation or anesthesia. Although US imaging is useful for detecting delicate ophthalmic lesions, such as cataract, retinal detachment, and asteroid hyalosis [15], a high-frequency transducer is required. Moreover, US cannot provide definitive information regarding bone invasion [15]. In addition to US, MRI is an excellent imaging modality for the eyes because it provides an excellent contrast image for soft tissues. Similar to US, MRI cannot elucidate information regarding bone alteration, including the orbital area, because of the lower number of hydrogen atoms in the bone for image production [15]. Furthermore, in animals, the MRI procedure requires a longer anesthetic duration, which could increase the anesthetic risk. In the last decade, CT has been considered the imaging technique of choice for the head and neck [15]. Because of the shorter acquisition time and volumetric image, CT has been applied to detect skull deformity, head trauma, and delineation of the tumor border in the head and neck region worldwide [15]. To evaluate the eye and adjacent structures on CT images, various factors such as skull type, age, sex, and body weight, which can affect normal eye parameters, should be considered [5,8]. However, the effects of these factors on CT images have never been reported in domestic cats. Therefore, this is the first report that utilized CT to compare normal ocular and orbital structures in the B and NB groups of cats.

In this study, all cats were mechanically restrained without sedation or general anesthesia. The advantages of this technique include reduced cost, client preference, and elimination of the risk of anesthesia, especially in B cats. Moreover, this technique may eliminate the effect of altered IOP on the eye parameters. The possibility of tranquilizers or anesthetic drugs affecting IOP has been reported. Dexmedetomidine, diazepam, and etomidate were reported to significantly reduce IOP [16,17], whereas ketamine could increase IOP [17]. Despite the fact that IOP and axial GL could fluctuate, the alteration of IOP did not affect GL during daytime in normal human eyes [18]. Although tranquilizers and anesthetic drugs can affect IOP directly through the action on the central diencephalic control center through facilitation or inhibition of aqueous production and drainage and through relaxation or contraction of extraocular and orbicularis oculi muscles, information on ocular biometry, including the size of adjacent eye structures on CT images, both before and after induction with these regimens, has never been reported. Therefore, further prospective studies comparing the eye parameters before and after chemical restraint are required.

The ocular biometry of all cats, B-cats, and NB-cats showed a significantly positive correlation between GL and age. In this study, GL increased concurrently with increasing age until the cat was 48 months old, before gradually decreasing GL dimension. This phenomenon is similar to that reported in humans [7]. Eye volume has been reported to have a significantly positive correlation with age until the age of 50 years before the gradual reduction of volume in some patients [7]. This reduction in the volume of the eye may be caused by the transformation of vitreous humor to a more fluid-like substance, with a subsequent decrease in IOP in senile patients. Therefore, the axial and sagittal lengths of the globe can be used to predict age in feline patients. However, for this, data accumulated from a large feline population is required.

In contrast to human reports [19], longer GL than GW was found in every group. This evidence might be caused by an interspecies difference in the skull of *Homo sapiens* and *Felis catus*. With regard to skull type, B-cats had a positive correlation between GW and age and had longer GW compared with NB-cats. However, ocular biometry observed by US revealed that sex, age, and body weight did not affect eye dimensions [20]. This discrepancy between studies may be due to different imaging modalities. CT can provide precise information using volumetric computational images.

In addition to the size of the globe, this study also provided information regarding the attenuation number of the anterior chamber, lens, and vitreous body of cats. The attenuation number did not differ for any factor. Therefore, the results of this study can be used as a reference value for prospective clinical diagnosis, for example, for cataract [21]. The 95% CIs of the pre-contrast, enhanced attenuation number of the anterior chamber, lens, and vitreous body were 10.90-14.05, 151.70-158.10, and 13.66-17.14 HU, respectively. These attenuation numbers revealed the same trends as those reported for canines [5]. Nevertheless, these results were obtained using a non-anesthetic and non-contrast-enhanced procedure. Therefore, the different attenuation numbers of intra-structural parameters due to the effects of anesthetic drugs and contrast agents should be considered. Prospective studies comparing the attenuation numbers both before and after sedation and/or general anesthesia, including contrast administration, should be conducted.

This study was also the first to provide orbital and retrobulbar information, including RD, in normal felines. Most external structures, both orbit and retrobulbar, appeared to correlate with body weight but not with age. This is different from a report on humans, which stated that orbital depth was positively correlated with age [22]. This discrepancy might have been caused by the age of the population, as the present study included only mature cats. The results from this study suggest that the greater the body weight of cats, the bigger the size of the skull, the longer the orbital depth, and RD. Information regarding orbital and retrobulbar sizes can be prospectively used as a preoperative prediction, especially in cats affected by vision-interfering retrobulbar mass. However, further studies with a large number of cats and varying diseases and different sizes of retrobulbar masses that may affect vision should be conducted.

This study had few limitations. A limited number of normal felines were included in this study because of the ethical regulation regarding use of animals for scientific work. A study with a large number of cats, including various breeds, and a comparison of ocular abnormalities, such as glaucoma, cataract, or other disease-induced size alterations, could have provided more information. Since this study was designed as a non-anesthetic examination, motion artifacts from animal movement were also counted as a limitation, and the results of this study could be applied only in animals without sedation or general anesthesia.

## Conclusion

The data regarding the globe, orbital, and retrobulbar areas in cats differed according to the skull type and age. The GW and orbital depth were affected by different skull types. GW was significantly wider in B cats, whereas the orbital depth was significantly greater in NB cats. In addition, GL, GW, and orbital diameter were affected by age showing a positive correlation. This information can be applied for ocular diagnosis and treatment, especially prosthetic surgical procedures.

## Authors’ Contributions

KY, NT, DD, and NC: Study conception and design. KY and NC: Acquisition of data. KY, CT, and NC: Analysis and interpretation of data. KY and NC: Drafting of manuscript. CT, NT, DD, and NC: Critical revision. All authors read and approved the final manuscript.

## References

[1] La Croix N.C, van der Woerdt A, Olivero D.K (2001). Nonhealing corneal ulcers in cats:29 Cases (1991-1999). J. Am. Vet. Med. Assoc.

[2] Featherstone H.J, Sansom J (2004). Feline corneal sequestra:A review of 64 cases (80 eyes) from 1993 to 2000. Vet. Ophthalmol.

[3] Gilger B.C, McLaughlin S.A, Whitley R.D, Wright J.C (1992). Orbital neoplasms in cats:21 Cases (1974-1990). J. Am. Vet. Med. Assoc.

[4] Cottrill N.B, Banks W.J, Pechman R.D (1989). Ultrasonographic and biometric evaluation of the eye and orbit of dogs. Am. J. Vet. Res.

[5] Salgüero R, Johnson V, Williams D, Hartley C, Holmes M, Dennis R, Herrtage M (2015). CT dimensions, volumes and densities of normal canine eyes. Vet. Rec.

[6] Morgan R.V, Daniel G.B, Donnell R.L (1994). Magnetic resonance imaging of the normal eye and orbit of the dog and cat. Vet. Radiol. Ultrasound.

[7] Igbinedion B.O, Ogbeide O.U (2013). Measurement of normal ocular volume by the use of computed tomography. Niger. J. Clin. Pract.

[8] Chiwitt C.L.H, Baines S.J, Mahoney P, Tanner A, Heinrich C.L, Rhodes M, Featherstone H.J (2017). Ocular biometry by computed tomography in different dog breeds. Vet. Ophthalmol.

[9] Mirshahi A, Shafigh S.H, Azizzadeh M (2014). Ultrasonographic biometry of the normal eye of the Persian cat. Aust. Vet. J.

[10] Raizada K, Rani D (2007). Ocular prosthesis. Cont. Lens Anterior Eye.

[11] Mohidin N, Chia J.Y, Saman M.N.M, Desai N (2013). Custom made ocular prosthesis at optometry clinic Universiti Kebangsaan Malaysia (UKM). J. Sains Kesihatan Malays.

[12] Jain R.K, Mehta R, Badve S (2010). Conjunctival squamous cell carcinoma due to ocular prostheses:A case report and review of literature. Pathol. Oncol. Res.

[13] Koch K.R, Trester W, Müller-Uri N, Trester M, Cursiefen C, Heindl L.M (2016). Ocular prosthetics Fitting, daily use and complications. Ophthalmology.

[14] Rao N.A (2013). Uveitis in developing countries. Indian J Ophthalmol.

[15] Penninck D, Daniel G.B, Brawer R, Tidwel A.S (2001). Cross-sectional imaging techniques in veterinary ophthalmology. Clin. Tech. Small Anim. Pract.

[16] Jaakola M.L, Ali-Melkkilä T, Kanto J, Kallio A, Scheinin H, Scheinin M (1992). Dexmedetomidine reduces intraocular pressure, intubulation responses and anaesthetic requirements in patients undergoing ophthalmic surgery. Br. J. Anaesth.

[17] Cunningham A.J, Barry P (1986). Intraocular pressure-physiology and implications for anesthetic management. Can. Anaesth. Soc. J.

[18] Wilson L.B, Quinn G.E, Ying G.S (2006). The relation of axial length and intraocular pressure fluctuations in human eyes. Invest. Ophthalmol. Vis. Sci.

[19] Salaam A.J, Aboje O.A, Danjem S.M, Tawe G, Salaam A.A (2016). Ocular biometry using computed tomography:In Jos, North Central Nigeria. Ophthalmol. Res.

[20] Gonçalves G.F, Pippi N.L, Raiser A.G, Mazzanti A (2000). Two-dimensional real-time ultrasonic biometry of ocular globe of dogs. Ciên. Rural.

[21] Wackenheim A, van Damme W, Kosmann P, Bittighoffer B Computed tomography in ophthalmology Density changes with orbital lesion. Neuroradiology.

[22] Chang J.T, Morrison C.S, Styczynski J.R, Mehan W, Sullivan S.R, Taylor H.O (2015). Pediatric orbital depth and growth:A radiographic analysis. J. Craniofac. Surg.

